# The effects of TMS stimulus direction on corticospinal and cortico‐cortical excitability of the lower limb in healthy adults

**DOI:** 10.14814/phy2.71144

**Published:** 2026-10-16

**Authors:** M. C. Hall, M. Zoghi, L. Perraton, S. Jaberzadeh

**Affiliations:** ^1^ Department of Physiotherapy, Non‐Invasive Brain Stimulation & Neuroplasticity Laboratory Monash University Frankston Victoria Australia; ^2^ Discipline of Physiotherapy, Institute of Health and Wellbeing Federation University Berwick Victoria Australia

**Keywords:** coil orientation, paired‐pulse, stimulus direction, threshold, tibialis anterior, TMS stimulus direction, transcranial magnetic stimulation

## Abstract

Transcranial magnetic stimulation (TMS) studies in upper limb muscles have shown that stimulus direction can influence corticospinal and intracortical excitability; however, limited evidence exists for lower limb muscles. This study investigated the effect of TMS stimulus direction on corticospinal and intracortical excitability of the tibialis anterior (TA) muscle using paired‐pulse TMS. Nineteen healthy adults (10 males, 9 females; mean age 41.8 ± 23 years) participated. Single‐ and paired‐pulse TMS was delivered using a figure‐of‐eight coil oriented to induce anterior‐to‐posterior (AP) or posterior‐to‐anterior (PA) current flow in the left motor cortex while recording motor evoked potentials (MEPs) from the right TA. Resting motor threshold (RMT), short‐interval intracortical inhibition (SICI), long‐interval intracortical inhibition (LICI), and intracortical facilitation (ICF) were assessed under both stimulus directions in a single session. No significant differences were identified between AP and PA stimulation for RMT, SICI, or LICI (*p* > 0.05). However, AP stimulation produced significantly greater ICF responses compared with PA stimulation (*p* = 0.027). AP and PA stimulus directions produced comparable RMT, SICI, and LICI measures in the TA muscle, although AP stimulation elicited greater ICF. These findings suggest facilitatory intracortical circuits may be more sensitive to stimulus direction than inhibitory pathways.

## 1 INTRODUCTION

Transcranial magnetic stimulation (TMS) is a noninvasive and painless technique that provides information about the excitability of the cortico‐cortical and corticospinal pathways in humans (Ziemann et al., 1996). TMS techniques are part of a growing body of literature concerned with the assessment of the primary motor cortex (M1) and its plasticity in a variety of conditions (Armijo‐Olivo, 2018).

The majority of TMS studies target the hand muscles due to their larger representation in the human homunculus (Christou et al., 2003; Sutherling et al., 1992), which may be due to the extensive use of fine motor tasks. Lower‐limb TMS research remains limited despite its relevance for gait, balance, rehabilitation, and neurological conditions affecting mobility. Understanding stimulus‐direction effects in the leg motor cortex is important because its deeper cortical representation and distinct geometry may alter intracortical recruitment compared with upper‐limb areas. Elicitation of TMS‐induced motor evoked potentials (MEPs) in leg muscles is harder as the map of the muscles in the lower limb is represented deeper within the cortex. The efficacy of the TMS volley diminishes with increasing distance between the TMS coil and the target tissue (Lotze et al., 2003). Moreover, according to the findings of Kesar et al. (2018) it has been suggested that there may be the possibility of fewer or weaker monosynaptic excitatory cortical projections that control lower limb muscles. Finally, larger scalp‐cortex distance (depth) compared to upper limb muscles is another reason for the main problem of evoking leg muscles with TMS.

Assessment of M1 excitability was performed using standard TMS procedures involving identification of the optimal stimulation site (“hotspot”) for the tibialis anterior, defined as the location producing the largest and most consistent MEPs during systematic coil mapping.

### 1.1 Theories on the mechanism of the effect of TMS stimulus direction

TMS stimulus direction affects the depth of penetration and strength of the induced electric field which may improve the elicitation of MEPs when aligned with the brain's fissures (Laakso et al., 2014). Contemporary models suggest that stimulus direction does not simply alter “current flow,” but instead biases recruitment of distinct neuronal populations and intracortical networks depending on cortical geometry and axonal orientation within M1 (Laakso et al., 2014). Consequently, different stimulus directions may preferentially recruit partially overlapping corticospinal and intracortical circuits, thereby influencing excitability measures and MEP generation.

Single‐pulse TMS delivered over M1 generates descending corticospinal volleys consisting of direct (D) and indirect (I) waves (Di Lazzaro et al., 1999). Paired‐pulse TMS outcomes are influenced by multiple methodological and physiological factors, including stimulation intensity, cortical state, coil‐to‐cortex distance, and background neural excitability, in addition to stimulus direction (Corp et al., 2021). D‐waves are thought to arise from direct activation of corticospinal axons, whereas I‐waves reflect transsynaptic activation of corticospinal neurons via excitatory intracortical interneuronal circuits (Di Lazzaro et al., 2013). Early and late I‐waves are believed to arise from partially distinct interneuronal networks and may be differentially sensitive to inhibitory and facilitatory modulation. Research has compared how Ia spindle afferents and corticospinal inputs connect to tibialis anterior (TA, a dorsiflexor) and soleus, but usually in terms of presence, strength, and modulation of connections—not as a simple count of connection density.

Importantly, although anterior–posterior (AP) and posterior–anterior (PA) orientations do not isolate specific I‐wave components or descending corticospinal volleys, they are known to bias recruitment toward different intracortical interneuronal networks within primary motor cortex (M1). PA stimulation tends to engage earlier‐recruited interneuronal circuits, whereas AP stimulation more strongly engages later‐recruited networks. This distinction is relevant because paired‐pulse measures such as short interval cortical inhibition (SICI), intracortical facilitation (ICF), and long‐interval cortical inhibition (LICI) depend on the excitability of these networks. Volz et al. (2015) examined directional effects on cortical connectivity but did not report motor system alterations. Similarly, Hamdan and Righetti (2005) investigated TBS‐related plasticity rather than paired‐pulse excitability. Although the present study does not directly measure I‐waves, AP and PA orientations are known to bias recruitment toward partially distinct intracortical interneuronal networks within M1. PA stimulation preferentially activates earlier‐recruited interneuronal circuits, whereas AP stimulation more strongly engages later‐recruited networks. These differences are relevant because paired‐pulse measures such as SICI, ICF, and LICI depend on the excitability of these intracortical circuits. Accordingly, stimulus direction may influence paired‐pulse outcomes indirectly through differential engagement of these networks, even though specific I‐wave components cannot be isolated in the present paradigm. While stimulus direction is an important determinant of corticospinal and intracortical excitability, the present findings also suggest that other methodological and physiological factors—including stimulation intensity, coil‐to‐cortex distance, cortical state, and background neural excitability—likely interact to influence paired‐pulse TMS outcomes. Consequently, stimulus direction alone may not fully explain variability in inhibitory and facilitatory responses across individuals or studies.

### 1.2 Studies on the effects of TMS stimulus direction

Fong et al. (2021) examined the effects of AP versus PA stimulus directions on SICI in the first dorsal interosseous (FDI) muscle. Similarly, Tugin et al. (2021) reported that a posterior‐lateral stimulus direction elicited greater SICI and short‐interval intracortical facilitation (SICF) than an anterior‐medial orientation in the FDI muscle; however, they concluded that conditioning stimulus intensity had a greater influence on these measures than stimulus direction itself.

Several upper‐limb studies have also reported greater SICI and LICI with AP compared with PA stimulation (Cirillo et al., 2018; Cirillo & Byblow, 2016; McMackin et al., 2024), supporting the concept that stimulus direction may bias activation toward different intracortical interneuronal populations. However, while AP stimulation may bias recruitment toward later‐activated intracortical networks, the specific interneuronal mechanisms cannot be directly inferred from paired‐pulse TMS.

Whether these findings generalize to lower‐limb muscles remains unclear, particularly given differences in cortical depth, interhemispheric fissure anatomy, and coil‐to‐cortex geometry. Hand et al. (2020) reported that a medio‐lateral (ML) stimulus direction over the TA produced lower resting motor thresholds and greater SICI than a PA orientation. Richter et al. (2013) similarly demonstrated lower thresholds using orientations anterior to the lateral‐medial plane, whereas Terao et al. (2000) found that a 90° stimulus direction produced the lowest thresholds compared with other orientations. However, these studies primarily focused on threshold or descending volley characteristics rather than paired‐pulse intracortical excitability.

Despite several lower‐limb studies examining non‐oblique or ML orientations, systematic comparison of oblique AP versus PA stimulus directions for both corticospinal and paired‐pulse intracortical excitability in the TA muscle remains limited. ML orientations were discussed only to contextualize prior lower‐limb studies; our study did not use ML stimulation. The present study therefore aimed to compare two commonly used oblique stimulus directions under pragmatic laboratory conditions without neuronavigation.

### 1.3 Aims and hypotheses

The main aim of this study was to investigate whether two commonly used oblique TMS stimulus directions, posterior–anterior (PA) and anterior–posterior (AP) orientations at 45° relative to the midline, produce systematic differences in corticospinal and cortico‐cortical excitability measures in the tibialis anterior muscle. Within this framework, SICI was included as the primary measure of intracortical inhibition due to its well‐established association with gamma‐amino‐butyric acid (GABA)a mediated inhibitory circuits.

In addition, ICF and LICI were examined to provide a more comprehensive characterization of cortico‐cortical excitability and to determine whether the effects of stimulus direction extend beyond a single inhibitory mechanism. These paradigms probe distinct, yet complementary, neurophysiological processes:

- SICI reflects fast‐acting inhibitory processes predominantly mediated by GABAa receptors.
- ICF is thought to reflect facilitatory glutamatergic mechanisms, particularly involving NMDA receptor activity.
- LICI assesses slower inhibitory processes associated with GABAb receptor‐mediated neurotransmission.

Including ICF and LICI as secondary outcomes therefore allows for a broader evaluation of the balance between cortical inhibition and facilitation, which is particularly relevant when investigating how TMS stimulus direction influences neural circuit recruitment. Given that PA and AP currents are known to differentially activate distinct intracortical pathways, assessing multiple measures of excitability provides a more nuanced interpretation of the underlying neurophysiological effects.

Accordingly, ICF and LICI were included as secondary outcome measures to explore whether any directional effects observed in SICI generalize to other excitatory and inhibitory pathways. Given the deeper cortical representation of the leg area, stimulus‐direction effects may differ from upper‐limb findings. Our results suggest that facilitatory circuits may show greater directional sensitivity than inhibitory circuits in lower‐limb M1.

We hypothesized that PA stimulation would produce lower RMTs than AP stimulation, and that AP stimulation would demonstrate greater intracortical inhibition relative to PA stimulation, consistent with preferential recruitment of later I‐wave–related interneuronal circuitry described in upper‐limb studies.

## 2 MATERIALS AND METHODS

### 2.1 Participants

Nineteen healthy adult volunteers were recruited for and completed the study (10 male, 9 female), mean age of 41.8 years (SD = 23), mean height of 176.4 cm (SD = 10.7) mean weight of 67.9 kg (SD = 16.6). No participants had neurological, psychological, or physical impairments, and none were on medications.

All participants completed the Adult Safety Screening Questionnaire (Opie et al., 2016) to determine their safety for TMS application. Participants gave informed written consent according to the declaration of Helsinki. Monash University's Human Research Ethics Committee (approval no. 21538) approved the experimental procedure. Participants were tested at approximately the same time of day (around 10 a.m.). All participants completed both PA and AP stimulus direction conditions within a single experimental session, with the order of conditions randomized across participants to minimize potential order or fatigue effects. All participants were asked to avoid caffeine and alcohol in the 24 h before testing. The testing session lasted approximately 40 min. All participants were stimulated over the left motor cortex to assess the right tibialis anterior muscle; however, handedness and lower‐limb dominance were not formally assessed or included in the analysis.

### 2.2 Electromyography

The participants were positioned in an upright chair, with their legs extended and their head supported by a headrest (Figure 1). Their right leg was exposed above the patella for electromyography (EMG) electrode placement. The skin over the TA muscle and patella was prepped with light sandpaper and alcohol swabs, as per Gilmore and Meyers (Gilmore & Meyers, 1983). Pre‐gelled self‐adhesive bipolar Ag/AgCl disposable surface electrodes were placed 1/3 of the distance between the tip of the fibula and the lateral malleolus and a ground electrode over the patella.

**FIGURE 1:**
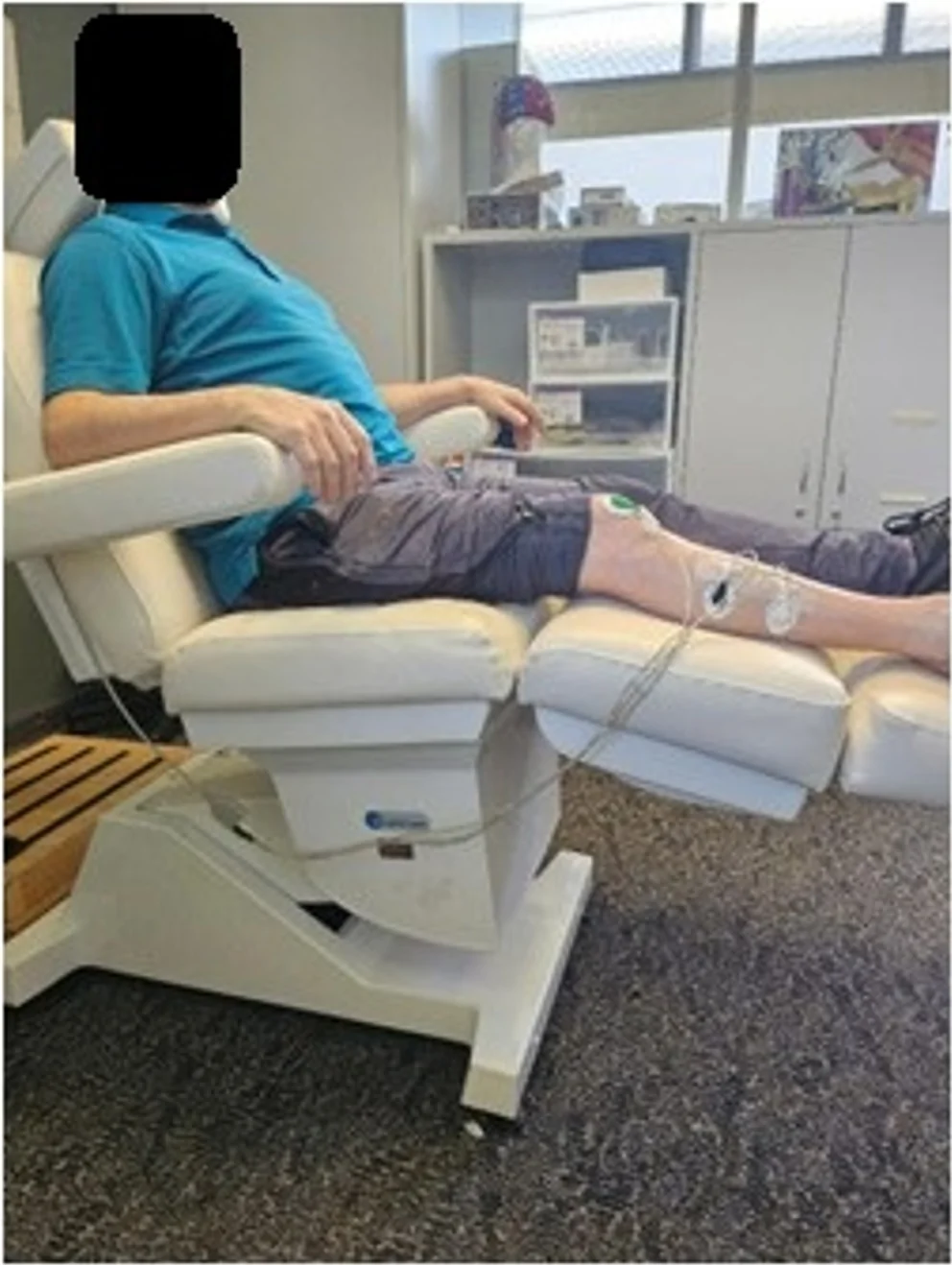
The testing position and location of electrode placement.

EMG signals were band‐pass filtered (10–500 Hz), amplified (×1000) and sampled at 1000 Hz, and collected on a PC running commercially available software (LabChart TM software, AD Instruments, Australia) via a laboratory analog‐digital interface (The PowerLab 8/30, ADInstruments, Australia) for later analysis.

### 2.3 Outcome measures

#### 2.3.1 Resting motor threshold

Single‐pulse magnetic stimuli were delivered using a MagPro R30 (MagVentrue, Denmark) stimulator with a butterfly MC‐PB70 figure‐of‐eight magnetic coil. Using the international 10–20 system, the vertex (Cz) point was identified and marked for use as a reference. The magnetic coil was placed over the left M1 area, contralateral to the target muscle. The coil was set at 45° to the midline and tangential to the scalp in the PA‐ML orientation (Figure 2a). The hotspot was defined as the location producing the largest and most consistent MEPs across ≥5 consecutive stimuli. The coil position was marked and monitored throughout the session to ensure consistency.

**FIGURE 2:**
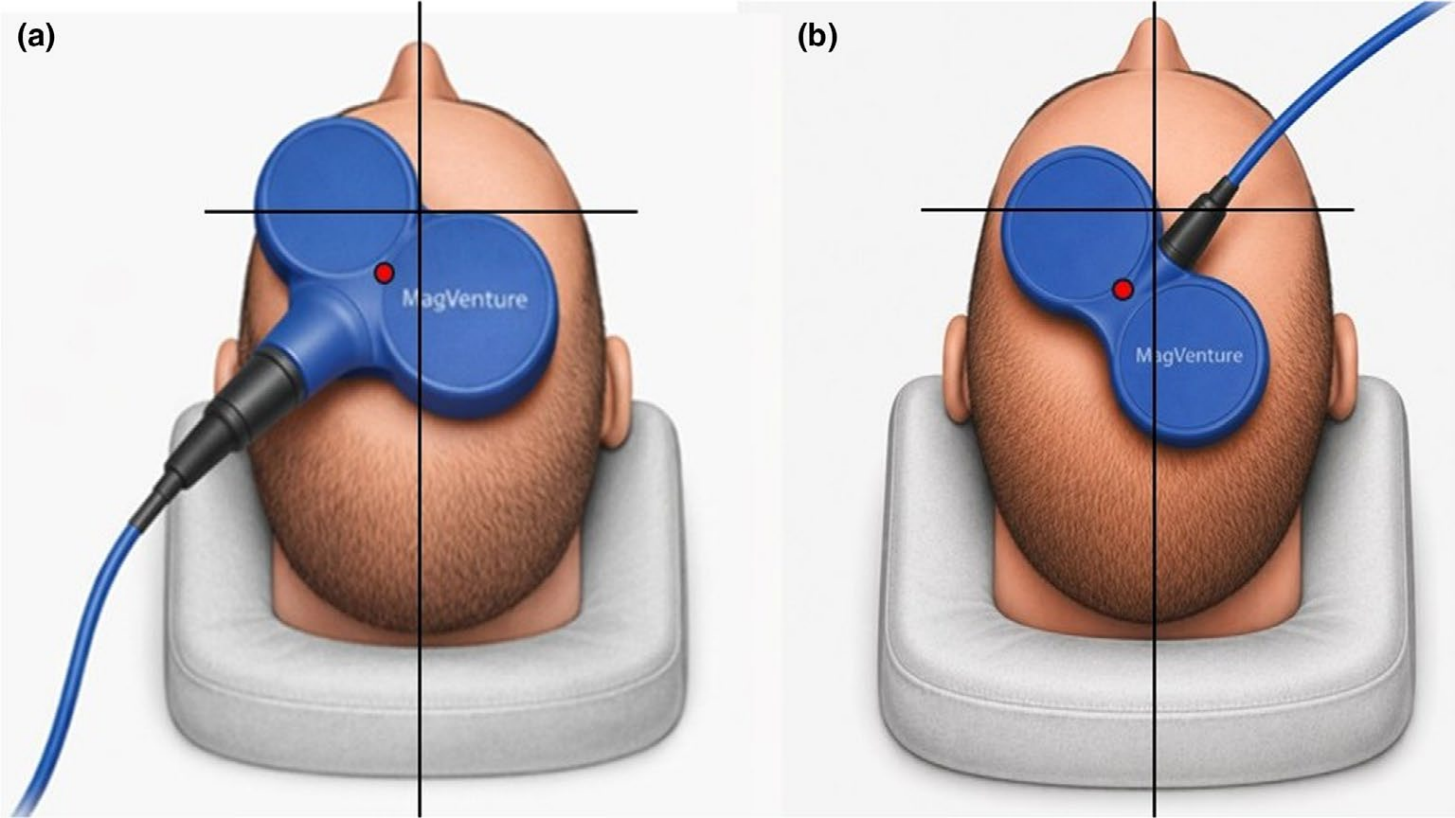
PA orientation (a), and AP orientation (b) The red dot indicates the “hotspot,” roughly 1 cm lateral and posterior to the vertex.

Resting motor threshold (RMT) was determined by a freeware program of TMS Motor Threshold Assessment Tool, MTAT 2.0, available at https://www.clinicalresearcher.org/software.htm, which utilizes a maximum likelihood parameter estimation through sequential testing without prior information (). In the case of RMT, a successful trial was determined by a MEP amplitude above 50 μV, indicated by a “yes” response. If this response was not observed, a “No” response was provided, and the MTAT software generated a new intensity displayed by the program. This process continued until the RMT was determined. A 0.5 mV target was selected to ensure reliable MEP detection in the tibialis anterior, which typically requires higher intensities due to deeper cortical representation.

#### 2.3.2 Intracortical excitability

##### Recording test MEP (Table 1)

**TABLE 1:** Stimulation intensities and outcomes.

Variable	AP stimulus direction	PA stimulus direction	Statistical outcome
Resting Motor Threshold (RMT) (% MSO)	61.16 ± 13.28	60.58 ± 11.26	*t*(18) = 0.481, *p* = 0.658
Test Intensity (TI) (% MSO)	No significant difference	No significant difference	*p* > 0.05
Test MEP Target Amplitude	~0.5 mV	~0.5 mV	Standardized across conditions
Conditioning Stimulus Intensity (SICI/ICF)	80% RMT	80% RMT	Matched relative intensity
SICI ISI	3 ms	3 ms	Fixed
ICF ISI	10 ms	10 ms	Fixed
LICI ISI	150 ms	150 ms	Fixed
SICI (% test MEP)	No significant difference	No significant difference	*p* > 0.05
LICI (% test MEP)	No significant difference	No significant difference	*p* > 0.05
ICF (% test MEP)	154.85 ± 9.92%	144.33 ± 9.95%	*F* = 4.914, *p* = 0.027
Inter‐trial Interval	6 s (fixed, no jitter)	6 s (fixed, no jitter)	Standardized
Number of Test MEPs	30	30	Per condition
Number of Paired‐Pulse Trials	90	90	Per condition
Coil Orientation	135° (AP)	45° (PA)	Relative to midline
Muscle State	Rest	Rest	Continuous EMG monitoring
Exclusion Criteria	Background EMG, artifact, MEP <0.1 mV	Same	Standardized
Participants	19 healthy adults	Same cohort	Within‐subject

The stimulation intensity was standardized using an MEP amplitude–based normalization approach, whereby intensities were adjusted to elicit a target motor evoked potential (MEP) amplitude of approximately 0.5 mV peak‐to‐peak at baseline. This approach places participants at a comparable point on the stimulus–response curve and helps reduce inter‐individual variability in corticospinal excitability, thereby improving the comparability of outcomes across participants. The test intensity for recording PA test MEPs was determined by increasing or decreasing the stimulator output intensity from 120% RMT until the mean peak‐to‐peak MEP amplitude across a short series of test pulses approximated 0.5 mV, acknowledging normal trial‐to‐trial variability in MEP amplitude (Bestmann, 2024; Ma et al., 2023). This intensity was called “Test Intensity” (TI). Thirty single‐pulse MEPs were recorded as test MEPs over the hotspot from the right TA muscle. All recordings were performed with the target muscle fully at rest to ensure methodological consistency across participants and to avoid the additional variability introduced by voluntary muscle contraction, including task‐dependent changes in corticospinal excitability and fluctuations in descending drive, which was continuously monitored using online EMG to ensure absence of pre‐activation. The TMS stimuli were applied every 6 s. As this was intentionally set to 0.5 mV in both conditions, no statistical analysis was performed on the effect of coil stimulus direction on test MEP (however, we did assess the differences of coil stimulus direction on RMT).

##### Recording conditioned PA MEPs

Thirty paired‐pulse MEPs were recorded by delivering a subthreshold conditioning stimulus (80% RMT) followed by a test stimulus at the TI with 3 ms or 10 ms ISI to be able to calculate the level of SICI (Nieminen et al., 2019) and ICF (Nikolov et al., 2020) respectively subsequently offline. All recordings were performed with the target muscle fully at rest, which was continuously monitored using online EMG to ensure absence of pre‐activation. These 60 paired‐pulse stimuli were administered randomly using a pre‐programmed protocol on the MagPro R30. A master spreadsheet was used to categorize each data point as either a SICI or ICF stimulus. The average of the paired‐pulse MEPs was compared to the average of the single‐pulse MEP and expressed as a percentage of the single‐pulse MEPs for the same stimulus orientation.

To assess long‐interval intracortical inhibition (LICI), 30 paired‐pulse MEPs were recorded using two suprathreshold stimuli delivered at the same test intensity, separated by an interstimulus interval of 150 ms (Opie et al., 2016). The amplitude of the conditioned MEP was expressed as a percentage of the unconditioned test MEP amplitude obtained for the same stimulus direction.

Each condition was treated as a distinct state, with the TI being determined for the AP TMS stimulus direction using the same hotspot as the PA orientation (as illustrated in Figure 2b). This process was repeated independently for both single and paired‐pulse AP test conditions, maintaining consistency by conducting each condition over the same hotspot (as summarized in Figure 3).

**FIGURE 3:**
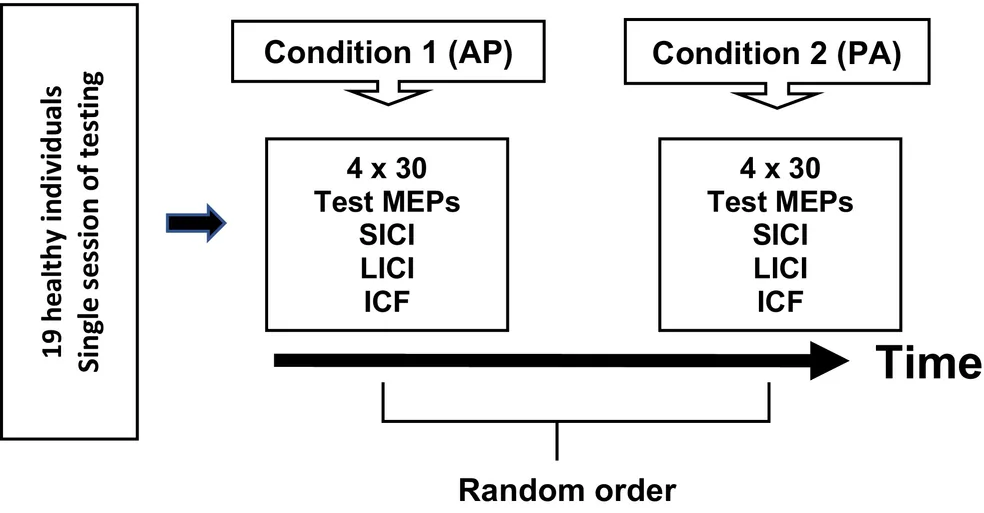
Experimental design. Nineteen healthy participants completed both AP and PA stimulus direction conditions within a single testing session. The order of AP and PA conditions was randomized across participants. For each stimulus direction, 30 single‐pulse test MEPs and 90 paired‐pulse conditioned MEPs were recorded to assess SICI, LICI, and ICF. AP, Anterior‐Posterior (stimulus direction); ICF, Intracortical Facilitation; LICI, Long‐interval Intracortical Inhibition; MEPs, Motor‐evoked potentials; PA, Posterior‐Anterior (stimulus direction); SICI, Short‐interval Intracortical Inhibition.

Inhibition appears when the test pulse follows the conditioning pulse by 1–7 ms, with peak inhibition around 2–3 ms. Facilitation emerges when ISI is longer, roughly 7–30 ms, with clear peaks around 10–15 ms. Long‐interval intracortical inhibition (LICI) in TMS is usually probed with interstimulus intervals (ISIs) between about 50–200 ms to index GABA Bmediated inhibition. Within this range, 100 and 150 ms have become common “standard” ISIs, but they are not equivalent.

### 2.4 Data processing and statistical analysis

During the experiment, 30 single pulse TMS were administered over the hotspot for both AP and PA orientations to record test MEPs. The investigator closely monitored each evoked response for any background artifacts, muscle contractions, and the amplitude of the MEP. Trials that exhibited artifacts, background EMG, or MEP amplitude below 0.1 mV were excluded and not included in the final calculations for each participant. This averaged 3–5 data points per participant.

For each participant and stimulus direction, MEP amplitude was quantified as the peak‐to‐peak EMG amplitude measured from the most negative to the most positive deflection following the TMS stimulus. The mean peak‐to‐peak amplitude of the test MEPs was then calculated for each participant and stimulus direction. Each conditioned MEP amplitude was then expressed as a percentage of that participant's mean test MEP amplitude for the corresponding stimulus direction. This approach retained trial‐level variability while accounting for within‐participant clustering in subsequent statistical modeling.

#### 2.4.1 Sample size

An a priori sample size estimate was performed using GPower *(v3.1)* to provide an approximate indication of the number of participants required to detect medium‐to‐large within‐subject effects, consistent with prior lower‐limb TMS studies. Formal power calculations for linear mixed‐effects models are not currently supported in GPower; therefore, this estimate should be interpreted as approximate rather than definitive.

#### 2.4.2 Statistical analysis

Prior to analysis, the data were assessed for normality of distribution with histogram curves. To analyze the effect of TMS stimulus direction on CCE, a linear mixed model analysis in SPSS v. 29 was used. The effects of TMS stimulus direction on ICF, SICI, and LICI were compared. The percentage of the paired‐pulse peak‐to‐peak as compared to the test MEP was considered the dependent variable. Coil orientation and test condition (ICF, SICI, or LICI) were considered fixed effects in the model. To control for the fact that we have multiple observations from each participant, we considered each subject as a random intercept.

A linear mixed‐effects model was selected because the study generated repeated trial‐level observations nested within participants across multiple paired‐pulse conditions and stimulus directions. This approach allowed retention of within‐subject variability while accounting for the non‐independence of repeated observations through participant‐specific random intercepts. Compared with averaging trials into single condition means, the LMM preserves more information and provides a more flexible framework for modeling repeated‐measures TMS data.

Finally, a paired‐sample *t*‐test was performed to analyze the effect of TMS stimulus direction on RMT. To aid interpretability, paired‐samples *t*‐tests were also performed for each outcome measure to confirm the direction and significance of observed effects.

## 3 RESULTS

To analyze the effect of TMS stimulus direction on cortico‐cortical excitability (CCE), a linear mixed‐effects model was performed in SPSS v29 (Table 2). The dependent variable was the conditioned MEP amplitude expressed as a percentage of the corresponding unconditioned test MEP. Fixed effects included stimulus direction (AP vs. PA), paired‐pulse condition (SICI, ICF, and LICI), and their interaction. Participant was included as a random intercept to account for repeated observations and within‐subject clustering of responses. Residual distributions were visually inspected to assess model assumptions. No significant difference was observed between AP and PA stimulus directions for resting motor threshold (AP: 61.16 ± 13.28% MSO; PA: 60.58 ± 11.26% MSO; *p* = 0.658). Similarly, no significant differences were observed in test intensities required to evoke ~0.5 mV MEPs between stimulus directions.

**TABLE 2:** LMM results.

ANOVA summary			
Effect	df	*F*	*p*
Condition	1, 18.05	29.405	<0.001
CoilOrientation	1, 17.94	0.203	0.658

Fixed effects estimates					
Term	Estimate	SE	df	*t*	*p*
Intercept	173.447	17.499	18.089	9.912	<0.001
Condition	−45.396	8.371	18.047	−5.423	<0.001
CoilOrientation	3.437	7.624	17.939	0.451	0.658

*Note*: The intercept corresponds to the (unweighted) grand mean; for each factor with *k* levels, *k*−1 parameters are estimated with sum contrast coding. Consequently, the estimates cannot be directly mapped to factor levels. Use estimated marginal means for obtaining estimates for each factor level/design cell or their differences.

### 3.1 Cortico‐cortical excitability

A linear mixed‐effects model demonstrated a weak but statistically significant interaction between stimulus direction and paired‐pulse condition (*F* = 3.794, *p* = 0.027). Post hoc analyses indicated that this interaction was driven primarily by ICF, with AP stimulation producing significantly greater facilitation than PA stimulation. No significant differences were observed for SICI or LICI (Figure 4).

**FIGURE 4:**
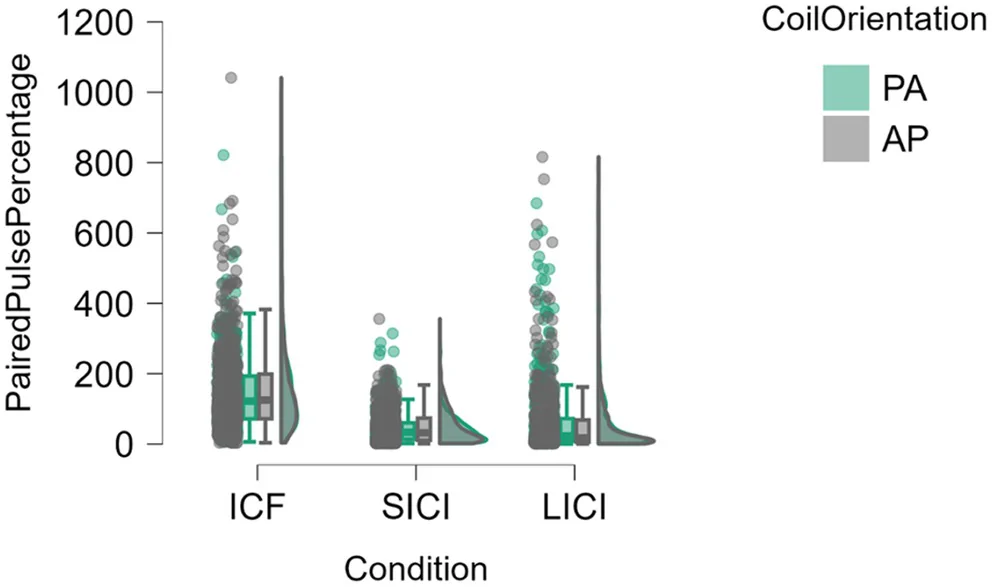
TMS stimulus orientation is significant for ICF (*p* = 0.027), but not SICI or LICI. Individual participant datapoints were included to illustrate the spread and variability of responses across participants.

## 4 DISCUSSION

### 4.1 Effects of stimulus direction on corticospinal excitability

This study examined whether two commonly used oblique TMS stimulus directions (AP and PA) produce different corticospinal and intracortical excitability outcomes in the tibialis anterior muscle. The results showed no significant differences in resting motor threshold (RMT) between AP and PA stimulation, indicating that both orientations recruited comparable corticospinal output under the present experimental conditions.

These findings contrast with some previous lower‐limb studies reporting threshold differences across stimulus directions (Hand et al., 2020; Richter et al., 2013). However, those studies used substantially different coil orientations (e.g., ML or LM), whereas the present study compared two oblique orientations positioned relative to the midline. Differences in coil orientation, cortical depth, and coil‐to‐cortex geometry likely contribute to variability across studies. Importantly, the deeper representation of the leg motor cortex may reduce directional sensitivity compared with upper‐limb areas, where AP/PA differences are more consistently observed.

Overall, the absence of RMT differences suggests that corticospinal membrane responsiveness in the lower‐limb motor cortex is relatively insensitive to AP versus PA orientation when intensities are standardized using amplitude‐based normalization.

### 4.2 Effects of stimulus direction on intracortical excitability

The primary intracortical finding was that AP stimulation produced significantly greater intracortical facilitation (ICF) compared with PA stimulation, whereas SICI and LICI did not differ between directions. This pattern indicates that facilitatory intracortical circuits may be more sensitive to stimulus direction than inhibitory circuits in the tibialis anterior representation.

Importantly, the present study does not infer specific interneuronal mechanisms or I‐wave components. Instead, the results reflect net intracortical excitability measured through standard paired‐pulse paradigms. The selective difference in ICF suggests that excitatory intracortical networks may be more directionally responsive than inhibitory networks in the lower‐limb motor cortex. This contrasts with some upper‐limb findings where AP stimulation often produces stronger inhibitory responses, highlighting potential differences in cortical geometry and network organization between hand and leg areas.

The lack of directional effects for SICI and LICI indicates that GABAa‐ and GABAb‐mediated inhibitory processes were largely comparable across orientations. Together, these results suggest that stimulus direction influences facilitatory intracortical processes more than inhibitory processes in the lower‐limb motor cortex under the present experimental conditions.

### 4.3 Limitations

Several limitations should be acknowledged. First, the study did not include ML or LM stimulus directions, which are often considered optimal for lower‐limb stimulation. Consequently, findings are restricted to the two oblique orientations tested.

Second, neuronavigation was not used. Although previous studies suggest that neuronavigation improves coil positioning accuracy and reduces variability (Cincotta et al., 2010; Herwig et al., 2001), the present study used consistent hotspot localisation procedures across conditions.

Third, the study did not directly assess MEP onset latency or descending corticospinal volleys. Therefore, interpretations regarding preferential recruitment of specific I‐wave components remain indirect and based on prior literature rather than direct physiological confirmation.

Fourth, considerable inter‐individual variability exists in TMS measures, and factors such as cortical anatomy, coil‐to‐cortex distance, hemispheric dominance, and corticospinal organization may influence directional sensitivity. The present study was not specifically designed or powered to investigate these participant‐level factors.

Finally, the sample size estimate was based on paired *t*‐test approximations rather than formal mixed‐model power calculations. Although the sample size was comparable with previous lower‐limb TMS studies, smaller directional effects may have gone undetected.

### 4.4 Future directions

Future studies incorporating MRI‐guided neuronavigation, individualized electric field modeling (e.g., SimNIBS), and structural imaging may provide greater insight into how cortical anatomy and electric field orientation influence recruitment of corticospinal and intracortical networks during lower‐limb TMS.

Additionally, studies incorporating ML, LM, AP, and PA stimulus directions within the same experimental paradigm would help clarify the relative contributions of stimulus direction to corticospinal and intracortical excitability in lower‐limb muscles.

## AUTHOR CONTRIBUTIONS

**M. C. Hall:** Conceptualization; data curation; formal analysis; investigation; methodology; project administration; resources; software. **M. Zoghi:** Conceptualization; formal analysis; methodology. **L. Perraton:** Project administration; supervision. **S. Jaberzadeh:** Conceptualization; methodology; project administration; software; supervision.

## FUNDING INFORMATION

The research presented in Physiological Reports was conducted without the receipt of any external funding. We hereby declare that there were no financial contributions, grants, sponsorships, or any form of financial support from any organization, institution, or entity for the completion of this study.

## CONFLICT OF INTEREST STATEMENT

The authors declare that there were no competing interests in the completion of this study. Throughout the entire research process, the authors involved in this research project did not have any financial, personal, or professional interests that could have influenced or biased the conduct or outcome of the study. There were no conflicts of interest that could have impacted the objectivity and integrity of the research presented in Physiological Reports. We affirm that all individuals who contributed to the development and dissemination of this study have been acknowledged appropriately, and their contributions were conducted with full transparency and integrity.

## ETHICS STATEMENT

This study was approved by the Monash University Human Research Ethics Committee (approval no. 21538) and conducted in accordance with the principles of the Declaration of Helsinki, except for pre‐registration in a publicly accessible database. Participants were healthy adult volunteers recruited with the support of Beleura Health Solutions. All participants received information about the study procedures and gave written informed consent before taking part.

Before testing, each participant completed the Adult Safety Screening Questionnaire to confirm they were suitable for transcranial magnetic stimulation (TMS). Participants had no neurological, psychological or physical impairments and were not taking medication. Stimulation was delivered using established TMS safety procedures. Both stimulus‐direction conditions (AP and PA) were completed in a single session of about 40 minutes. No adverse events were reported by any subjects.

## ARTIFICIAL INTELLIGENCE DISCLOSURE STATEMENT

OpenAI's ChatGPT (GPT‐5.5) was used during manuscript preparation in 2026 to assist with language refinement, grammatical editing, sentence restructuring, and organization of document flow. The AI tool was also used to assist in drafting and revising portions of the Introduction, Discussion, and Abstract sections to improve clarity, readability, and scientific expression. The authors directed all prompts provided to the AI system, critically reviewed all generated content, and independently verified the scientific accuracy, interpretation, terminology, statistical reporting, and referenced literature. No data analysis, data interpretation, or autonomous scientific conclusions were performed by the AI technology. All final decisions regarding manuscript content, structure, and conclusions were made solely by the authors, who accept full responsibility for the integrity and accuracy of the work.

## Data Availability

The data that support the findings of this study are available from the corresponding author, MH, upon reasonable request.
